# Measuring antibiotic availability and use in 20 low- and middle-income countries

**DOI:** 10.2471/BLT.19.241349

**Published:** 2020-01-31

**Authors:** Rebecca Knowles, Mike Sharland, Yingfen Hsia, Nicola Magrini, Lorenzo Moja, Amani Siyam, Elizabeth Tayler

**Affiliations:** a Nuffield Department of Population Health, University of Oxford, Oxford, England.; b Paediatric Infectious Disease Research Group, St George’s University London, London, England.; c Department of Essential Medicines and Health Products, World Health Organization, Geneva, Switzerland.; d Data Analytics and Delivery for Impact Division, World Health Organization, Geneva, Switzerland.; e Global Coordination and Partnerships Group, Antimicrobial Resistance Division, World Health Organization, Avenue Appia 20, Geneva 27, 1211, Switzerland.

## Abstract

**Objective:**

To assess antibiotic availability and use in health facilities in low- and middle-income countries, using the service provision assessment and service availability and readiness assessment surveys.

**Methods:**

We obtained data on antibiotic availability at 13 561 health facilities in 13 service provision assessment and 8 service availability and readiness assessment surveys. In 10 service provision assessment surveys, child consultations with health-care providers were observed, giving data on antibiotic use in 22 699 children. Antibiotics were classified as access, watch or reserve, according to the World Health Organization’s AWaRe categories. The percentage of health-care facilities across countries with specific antibiotics available and the proportion of children receiving antibiotics for key clinical syndromes were estimated.

**Findings:**

The surveys assessed the availability of 27 antibiotics (19 access, 7 watch, 1 unclassified). Co-trimoxazole and metronidazole were most widely available, being in stock at 89.5% (interquartile range, IQR: 11.6%) and 87.1% (IQR: 15.9%) of health facilities, respectively. In contrast, 17 other access and watch antibiotics were stocked, by fewer than a median of 50% of facilities. Of the 22 699 children observed, 60.1% (13 638) were prescribed antibiotics (mostly co-trimoxazole or amoxicillin). Children with respiratory conditions were most often prescribed antibiotics (76.1%; 8972/11 796) followed by undifferentiated fever (50.1%; 760/1518), diarrhoea (45.7%; 1293/2832) and malaria (30.3%; 352/1160).

**Conclusion:**

Routine health facility surveys provided a valuable data source on the availability and use of antibiotics in low- and middle-income countries. Many access antibiotics were unavailable in a majority of most health-care facilities.

## Résumé

**Objectif:**

Mesurer la disponibilité et l'usage des antibiotiques au sein des établissements médicaux dans les pays à faible et moyen revenu, en recourant à des enquêtes d'évaluation des prestations de service, ainsi que de la disponibilité et de l'état de préparation.

**Méthodes:**

Nous avons obtenu des données sur la disponibilité des antibiotiques dans 13 561 établissements médicaux dans le cadre de 13 enquêtes d'évaluation des prestations de service et 8 enquêtes d'évaluation de la disponibilité et de l'état de préparation. Pour 10 de ces 13 enquêtes d'évaluation des prestations de service, ce sont les consultations en pédiatrie impliquant du personnel soignant qui ont été observées, ce qui a permis d'accéder à des données sur l'usage des antibiotiques chez 22 699 enfants. Les antibiotiques ont été répartis en trois groupes, conformément au principe AWaRe mis en place par l'Organisation mondiale de la Santé : antibiotiques dont l'accessibilité est essentielle (Access), antibiotiques à utiliser sélectivement (Watch) et antibiotiques de réserve (Reserve). Le pourcentage d'établissements médicaux possédant des antibiotiques spécifiques ainsi que la proportion d'enfants ayant reçu des antibiotiques pour des syndromes cliniques clés ont été estimés dans différents pays.

**Résultats:**

Les enquêtes ont évalué la disponibilité de 27 antibiotiques (19 de la catégorie Access, 7 de la catégorie Watch, 1 non catégorisé). Le cotrimoxazole et le métronidazole étaient les plus répandus, présents dans 89,5 % des stocks (écart interquartile, EI : 11,6 %) et 87,1 % (EI : 15,9 %) des établissements médicaux. En revanche, 17 autres antibiotiques appartenant aux catégories Access et Watch étaient en stock chez moins de la médiane de 50 % des établissements. Sur les 22 699 enfants observés, 60,1 % (13 638) se sont vu prescrire des antibiotiques (principalement du cotrimoxazole ou de l'amoxicilline). Ce sont les enfants présentant des affections respiratoires qui ont le plus souvent été traités aux antibiotiques (76,1 % ; 8972/11 796), suivis par ceux souffrant d'une fièvre indifférenciée (50,1 % ; 760/1518), d'une diarrhée (45,7 % ; 1293/2832) et de la malaria (30,3 % ; 352/1160).

**Conclusion:**

Les enquêtes de routine menées dans les établissements médicaux constituent une précieuse source d'informations sur la disponibilité et l'usage des antibiotiques dans les pays à faible et moyen revenu. De nombreux antibiotiques dont l'accessibilité est essentielle (Access) étaient absents chez la plupart des établissements médicaux.

## Resumen

**Objetivo:**

Evaluar la disponibilidad y el uso de antibióticos en los centros sanitarios de los países de ingresos bajos y medios, mediante la evaluación sobre la prestación de servicios y las encuestas de evaluación sobre la disponibilidad y la preparación de los servicios.

**Métodos:**

Se obtuvieron datos sobre la disponibilidad de antibióticos en 13 561 centros sanitarios en 13 encuestas de evaluación sobre la prestación de servicios y en 8 encuestas de evaluación sobre la disponibilidad y la preparación de los servicios. En 10 encuestas de evaluación sobre la prestación de servicios se observaron consultas de niños con proveedores de atención sanitaria, lo que permitió obtener datos sobre el uso de antibióticos en 22 699 niños. La herramienta AWaRe de la Organización Mundial de la Salud clasificó los antibióticos como de acceso, vigilancia o reserva. Se estimó el porcentaje de centros de atención sanitaria de todos los países que disponían de antibióticos específicos y la proporción de niños que recibían antibióticos para los principales síndromes clínicos.

**Resultados:**

Las encuestas evaluaron la disponibilidad de 27 antibióticos (19 de acceso, 7 de vigilancia, 1 sin clasificar). El cotrimoxazol y el metronidazol fueron los antibióticos con mayor disponibilidad, ya que se encontraban en existencias en el 89,5 % (rango intercuartil, IQR: 11,6 %) y el 87,1 % (IQR: 15,9 %) de los centros de salud, respectivamente. En cambio, otros 17 antibióticos de acceso y vigilancia estaban almacenados en menos de una mediana del 50 % de los centros. De los 22 699 niños observados, al 60,1 % (13 638) se les recetaron antibióticos (principalmente cotrimoxazol o amoxicilina). A los niños con afecciones respiratorias se les recetó con mayor frecuencia antibióticos (76,1 %; 8 972/11 796), seguidos por aquellos con fiebre indiferenciada (50,1 %; 760/1 518), diarrea (45,7 %; 1 293/2 832) y malaria (30,3 %; 352/1 160).

**Conclusión:**

Las encuestas de rutina en los centros sanitarios constituyeron una valiosa fuente de datos sobre la disponibilidad y el uso de antibióticos en los países de ingresos bajos y medios. En la mayoría de los centros de atención sanitaria no se disponía de muchos antibióticos de acceso.

## ملخص

**الغرض:**

تقييم مدى توافر المضادات الحيوية واستخدامها في المرافق الصحية في البلدان ذات الدخل المنخفض والمتوسط، وذلك باستخدام مسوح لتقييم كل من الاستعداد، ومدى توافر الخدمات، وتقديم الخدمات.

**الطريقة:**

لقد حصلنا على بيانات حول توفر المضادات الحيوية في 13561 مرفقًا صحيًا في 8 مسوح لتقييم الاستعداد ومدى توافر الخدمات و13 دراسة استقصائية لتقديم الخدمات. وفي 10 مسوح لتقييم تقديم الخدمات، لوحظت مشاورات الأطفال مع مقدمي الرعاية الصحية، مما وفر بيانات عن استخدام المضادات الحيوية على 22699 طفلًا. تم تصنيف المضادات الحيوية كمضادات حيوية يجب أن تكون متاحة طوال الوقت بسعر مناسب (ACCESS)، أو مضادات حيوية يوصى بها كاختيار أول أو ثان (WATCH)، أو مضادات حيوية تستخدم كحل أخير (RESERVE)، وفقًا لفئات AWaRe التابعة لمنظمة الصحة العالمية. تم تقدير النسبة المئوية لمرافق الرعاية الصحية في جميع البلدان التي بها مضادات حيوية متاحة بالإضافة إلى نسبة الأطفال الذين يتلقون المضادات الحيوية للمتلازمات السريرية الرئيسية.

**النتائج:**

قيّمت المسوح مدى توافر 27 من المضادات الحيوية (19 مضادًا حيويًا يجب أن يكون متاحًا طوال الوقت بسعر مناسب، و7 مضادات حيوية يوصى بها كاختيار أول أو ثان، وواحد غير مصنف). كان كل من كوتريموكسازول وميترونيدازول متاحين على أوسع نطاق، حيث كانا متوفران بنسبة 89,5% (المدى الربيعي، IQR: 11,6%) و87,1% (IQR: 15,9%) من المرافق الصحية، على التوالي. وفي المقابل، تم تخزين 17 مضاداً حيوياً آخر ليكون متاحًا طوال الوقت بسعر مناسب وللاستخدام كاختيار أول أو ثان، بأقل من متوسط 50% من المرافق. من بين الـ 22699 طفلًا الذين تمت ملاحظتهم، وُصف لـ 60,1% منهم مضادات حيوية (أغلبها من كوتريموكسازول أو الأموكسيسيلين). غالبًا ما يتبع وصف المضادات الحيوية للأطفال المصابين بأمراض في الجهاز التنفسي (76.1%؛ 8972/11796) حمى غير متمايزة (50,1%؛ 760/1518)، وإسهال (45,7%؛ 1293/2832) وملاريا (30,3%؛ 352/1160).

**الاستنتاج:**

قدمت المسوح الروتينية للمرافق الصحية مصدرًا قيمًا للبيانات حول مدى توفر المضادات الحيوية واستخدامها في البلدان ذات الدخل المتوسط والمنخفض. والعديد من المضادات الحيوية التي يجب أن تكون متاحة طوال الوقت بسعر مناسب، لم تكن متوفرة في غالبية مرافق الرعاية الصحية.

## 摘要

**目的:**

旨在评估中低收入国家医疗机构中抗生素的可用性和使用情况，采用服务提供评估以及服务可用性和准备情况评估调查。

**方法:**

我们通过 13 项服务提供评估以及 8 项服务可用性和准备情况评估调查，获得了 13 561 个医疗机构的抗生素可用性数据。在 10 项服务提供评估调查中，观察了医疗护理人员参与的儿童会诊，提供了 22 699 名儿童的抗生素使用数据。根据世界卫生组织的知晓 (AWaRe) 类别，抗生素被分为可广泛使用、谨慎使用或保留使用。估算了可获得特定抗生素的国家中的医疗机构所占的百分比以及因主要临床综合征而接受抗生素的儿童比例。

**结果:**

调查评估了 27 种抗生素的可用性（19 种可广泛使用、7 种谨慎使用、1 种未分类）。复方新诺明和甲硝唑的使用最为广泛，医疗机构中库存率分别为 89.5％（四分位距，IQR：11.6％）和 87.1％（IQR：15.9%）。相比之下，其他 17 种可广泛使用、谨慎使用的库存抗生素，在医疗机构中不到中位数的 50％。在观察到的 22 699 名儿童中，有 60.1％ (13 638) 的儿童开了抗生素（主要是复方新诺明或阿莫西林）。患有呼吸道疾病的儿童最常使用抗生素（76.1％；8972/11 796），其次是无特异性发烧（50.1％；760/1518）、腹泻（45.7％；1293/2832）和疟疾（30.3％；352/1160）。

**结论:**

常规医疗机构调查提供了有关中低收入国家抗生素的可用性和使用情况的重要数据来源。在大多数医疗护理机构中，许多可广泛使用的抗生素都不可用。****

## Резюме

**Цель:**

Оценить доступность антибиотиков и их использование в учреждениях здравоохранения в странах с низким и средним уровнем дохода путем применения опросов для оценки предоставления услуг, их доступности и готовности к их оказанию.

**Методы:**

Авторы получили данные о доступности антибиотиков в 13 561 учреждении здравоохранения на основании 13 оценок предоставления услуг и 8 опросов для установления доступности услуг и готовности к их оказанию. В 10 опросах с целью оценки предоставления услуг проводилось наблюдение за детскими медицинскими консультациями, что позволило получить данные по применению антибиотиков у 22 699 детей. Антибиотики классифицировались как доступные, применяемые под наблюдением или с ограничениями согласно категориям AWaRe, принятым ВОЗ. Была проведена оценка процентной доли учреждений здравоохранения в разных странах, в которых были доступны конкретные антибиотики, и доли детей, получавших антибиотики по причине основных клинических синдромов.

**Результаты:**

В ходе исследования была оценена доступность 27 антибиотиков (19 доступных, 7 используемых под наблюдением, 1 неклассифицированный). Наиболее доступными были котримоксазол и метронидазол, их запас присутствовал в 89,5% (межквартильный диапазон МКД: 11,6%) и 87,1% (МКД: 15,9%) медицинских учреждений соответственно. Напротив, запасы 17 других доступных и применяемых под наблюдением антибиотиков имелись менее чем в 50% (медианное значение) всех учреждений. Из 22 699 наблюдаемых детей 60,1% (13 638) получали антибиотики по назначению врача (в основном котримоксазол или амоксициллин). Чаще всего антибиотики прописывали детям с респираторными заболеваниями (76,1%; 8972/11 796), а затем при недифференцированной лихорадке (50,1%; 760/1518), диарее (45,7%; 1293/2832) и малярии (30,3%; 352/1160).

**Вывод:**

Регулярные опросы о работе медицинских учреждений позволили получить ценные данные относительно доступности и использования антибиотиков в странах с низким и средним уровнем дохода. В большинстве медицинских учреждений многие доступные антибиотики отсутствовали.

## Introduction

The reliable availability of affordable, high-quality antibiotics remains a major global concern.^1^^,^^2^ Antibiotics are vital for preventing and treating bacterial infection, without which the risk of surgery becomes greater, managing noncommunicable disease becomes more difficult and universal health coverage becomes less attainable. Sustainable development goal (SDG) 3.8 includes the achievement of, “access to safe, effective, quality and affordable essential medicines and vaccines for all.”^3^ However, although ensuring universal access to antimicrobials can save millions of lives,^4^ excessive and inappropriate use must be limited to avoid the development of antimicrobial resistance.

An insight into the specific types of antibiotics available and used in different countries is vital. When the correct medication is not available, it may be substituted by an alternative, such as a broad-spectrum antibiotic, and patients may buy over-the-counter medicines that could be falsified or of a poor quality. These alternatives can be less effective, have more adverse effects and could drive the development of antimicrobial resistance.^5^^,^^6^ The reduced effectiveness of antimicrobials and the increasing burden of antimicrobial resistance are particularly problematic in low- and middle-income countries, where multidrug-resistant pathogens (e.g. *Escherichia coli* and *Salmonella* species) are common.^6^^–^^9^

Although it may be unrealistic and undesirable to achieve universal access to all antibiotics at all health facilities, it should be possible to ensure consistent access to key antibiotics. The AWaRe (access, watch, reserve) antibiotics categories (Box 1 and Table 1; both available at: http://www.who.int/bulletin/volumes/98/3/19-241349) of the World Health Organization’s (WHO’s) 2019 list of essential medicines includes a core set of antibiotics that should be available everywhere (i.e. access antibiotics) because they are the first and second choice for treating common or severe clinical syndromes.^10^^,^^12^ These antibiotics are generally narrow-spectrum agents with a low risk of resistance selection and of adverse effects. The other two AWaRe categories are: (i) watch antibiotics, which have a higher risk of toxicity or resistance development; and (ii) reserve antibiotics, which should be used as a last resort in specific clinical situations and whose effectiveness should be preserved.^10^^,^^12^

Box 1 **AWaRe antibiotics categories**

In 2017, the World Health Organization’s Expert Committee on the Selection and Use of Essential Medicines undertook a comprehensive review of antibacterials on the Model List of Essential Medicines that are used to treat common, priority infectious syndromes.^10^^,^^11^ The antibiotics included on the list were revised and listed as first- or second-choice treatments for specific indications. The Committee also proposed assigning antibiotics to three groups: access, watch and reserve antibiotics (i.e. the AWaRe categories).

• Access antibiotics are first- and second-choice antibiotics for the empirical treatment of most common infectious syndromes;

• Watch antibiotics include classes of antibiotics that have a higher potential for the development of resistance and whose use as first- or second-choice treatment should be limited to a small number of syndromes or patient groups; and

• Reserve antibiotics are antibiotics that should be used mainly as treatments of last resort.

The AWaRe categories consider the need to ensure access to necessary antibiotics, the need for effective antimicrobial stewardship and the impact of different antibiotics on antimicrobial resistance. They provide a useful tool for identifying which antibiotics to monitor and for informing procurement and supply policies.

**Table 1:** The AWaRe (access, watch, reserve) antibiotics categories of the World Health Organization’s 2019 list of essential medicines^10^

Antibiotics category^a^			
Access	Watch	Reserve	Other^b^
**Antibiotics assessed in the surveys^c^**			
Amoxicillin Ampicillin Amoxicillin with clavulanic acid Benzathine Benzylpenicillin Cloxacillin Chloramphenicol Clindamycin Doxycycline Gentamycin Metronidazole Procaine benzylpenicillin Streptomycin Sulfamethoxazole and trimethoprim (co-trimoxazole) Tetracycline Cefalexin Penicillin	Ciprofloxacin Third-generation cephalosporins, with or without a β-lactamase inhibitor (i.e. cefixime, ceftriaxone and cefotaxime) Macrolides (i.e. azithromycin, clarithromycin and erythromycin)	None	Kanamycin
**Antibiotics not assessed in the surveys^c^**			
Nitrofurantoin Phenoxymethylpenicillin Spectinomycin First-generation cephalosporins other than cefalexin	Quinolones and fluoroquinolones other than ciprofloxacin (e.g. levofloxacin, moxifloxacin and norfloxacin) Third-generation cephalosporins, with or without a β-lactamase inhibitor, other than cefixime, ceftriaxone and cefotaxime (e.g. ceftazidime) Glycopeptides (e.g. teicoplanin and vancomycin) Antipseudomonal penicillins with a β-lactamase inhibitor (e.g. piperacillin with tazobactam) Carbapenems (e.g. meropenem and imipenem with cilastatin) Penems (e.g. faropenem) Second-generation cephalosporins	Aztreonam Fourth-generation cephalosporins (e.g. cefepime) Fifth-generation cephalosporins (e.g. ceftaroline) Polymyxins (e.g. polymyxin B and colistin) Fosfomycin (intravenous) Oxazolidinones (e.g. linezolid) Daptomycin	None

^a^ Access antibiotics are the first and second choice for treating common or severe clinical syndromes, watch antibiotics have a higher risk of toxicity or resistance development and reserve antibiotics should be used as a last resort to preserve their effectiveness (Box 1).

^b^ This column lists only the one unclassified antibiotic that was included in surveys.

^c^ Surveys include the service availability and readiness assessments and service provision assessments.

Monitoring the progress of efforts to address antimicrobial resistance requires data on not only resistance patterns, but also on the availability and use of antibiotics, and how they are changing. External surveys of health facilities carried out as part of service provision assessments and service availability and readiness assessments include data on antibiotic availability, and potentially provide countries with an overview of the antibiotics available locally (Box 2; available at: http://www.who.int/bulletin/volumes/98/3/19-241349).^13^^,^^14^ Other approaches to monitoring the availability of drugs (including antibiotics) are the medicines monitoring tools used to generate data for monitoring SDGs (currently used in five countries and being extended to others).^15^ Several countries also track stock-outs of medicines (usually including some antibiotics) at individual facilities as a performance metric for health systems.

Box 2 **Service availability and readiness assessment surveys and service provision assessment surveys**

Service availability and readiness assessment surveys and service provision assessment surveys are health facility surveys that assess the availability and readiness of different health services in a country with reference to accepted standards of care. In addition, service provision assessment surveys also include observations of patient care and evaluate client satisfaction with service delivery. Both survey tools generate indicators of service availability and readiness that provide reliable and regular information on: (i) service delivery processes and provisions, such as the availability of key human and infrastructure resources, basic equipment, basic amenities, essential medicines and diagnostic capacities; and (ii) the readiness of facilities to provide basic health-care interventions, such as family planning, child health services, basic and comprehensive emergency obstetric care, and the treatment of HIV infection, tuberculosis, malaria and noncommunicable diseases. Currently, service availability and readiness assessment surveys are being implemented in 32 countries and service provision assessment surveys are being implemented in 17. As the average time between surveys is 2 to 3 years, they are not intended to replace routine supervision and monitoring. Instead, they collect information that can provide an external validation of whether health systems are functioning as reported. In particular, they provide an ideal way of verifying service standards in countries where accreditation and certification systems are undergoing revision and improvement.

The aim of this study was to determine whether external assessments of health facilities in low- and middle-income countries can provide data on antibiotic availability and use in general, and on the availability of key antibiotics in particular. To do this, we used data from service provision assessment and service availability and readiness assessment surveys in low- and middle-income countries to calculate the proportion of health facilities that held stocks of core antibiotics in each country, and the proportion of children prescribed antibiotics for key clinical syndromes.

## Methods

The service provision assessment includes a cross-sectional, health facility survey developed by ICF International Inc. under the Demographic and Health Surveys (DHS) programme funded by the United States Agency for International Development.^13^ The service availability and readiness assessment surveys are conducted by WHO using very similar methods (Box 2 and Table 2; both available at: http://www.who.int/bulletin/volumes/98/3/19-241349).^16^ Full details of the surveys’ procedures, methods and questionnaires are available online.^17^^,^^18^ For both types of survey, health facilities were selected in each country from national facility lists, which included private, non-profit and faith-based hospitals, and health centres.^19^ The surveys used nationally representative samples of the formal health system in all countries except Haiti, Malawi, Mauritania, Namibia, Rwanda and Uganda, where all or almost all facilities were included.

**Table 2:** Comparison of service provision assessment and service availability and readiness assessment surveys

Characteristic	Survey	
	Service provision assessment^13^	Service availability and readiness assessment^16^
Survey conducted by	DHS using the USAID–WHO inventory questionnaire	WHO and USAID
Background	The survey was developed by updating the method used in service availability and readiness assessment surveys to cover more areas and to give a more comprehensive overview. The topics covered include equipment, amenities, essential medicines, diagnostic capacity and the readiness of health facilities to provide basic health-care interventions for family planning, child health, obstetric care, HIV infection, tuberculosis, malaria and noncommunicable diseases	The survey was developed through a joint WHO–USAID collaboration. The health facility assessment tool was designed to assess and monitor service availability and the readiness of a country’s health sector, and to generate evidence to support planning and management. The topics covered include equipment, amenities, essential medicines, diagnostic capacity and the readiness of health facilities to provide basic health-care interventions for family planning, child health, obstetric care, HIV infection, tuberculosis, malaria and noncommunicable diseases
Survey elements	(i) Inventory questionnaire (including data on antibiotic availability); (ii) observation protocols and interviews with clients leaving facilities about antenatal care, family planning and sick children (including data on antibiotic use in children); and (iii) Service provision assessment health worker and health-care provider interview questionnaire	Inventory questionnaire (including data on antibiotic availability)
Method and health facilities included	(i) The survey typically includes 400–700 facilities (surveys can be carried out either as a census or as a representative sample of health facilities) selected from the country’s master facility list; (ii) surveys are typically conducted by 10–15 teams, each comprising 3–4 interviewers (mostly health workers); and (iii) interviewers collect data from the people in charge or the most knowledgeable people at each facility using the inventory questionnaire, observe consultations and interview clients leaving facilities	These surveys use the same method as service provision assessment surveys, except that consultations are not observed and clients are not interviewed on leaving facilities
Data availability	Available online for each country	WHO has all data

DHS: Demographic and Health Surveys; HIV: human immunodeficiency virus; USAID: United States Agency for International Development; WHO: World Health Organization.

Each assessment was based on an inventory questionnaire completed by trained interviewers from WHO or the DHS programme during a visit to the health facility and provided an external validation of the facility’s functioning.^20^ Antibiotics were audited to determine if they were in stock at each facility on the interview day. In most service provision assessment surveys, interviewers also observed child consultations to assess adherence to standards of care provision and antibiotic prescription.

Details of the survey design are available online for most countries.^13^^,^^21^ In addition, data sets for the service provision assessments are publicly available from the DHS programme and service availability and readiness assessment data sets are available from WHO. The surveys included in our study sample were those: (i) for which microdata were available (rather than just survey reports); (ii) that had been conducted after 2000 (studies completed between 1997 and 2000 were less comparable because different survey instruments were used); and (iii) that provided the most recent data set available for the country (available in the data repository).^22^ For countries where several surveys had been performed, we used the latest survey that provided data on antibiotic use.

### Antibiotic availability and use

An antibiotic was considered available at a facility if the medications in stock on the assessment day were within their usage dates and were, therefore, available for patients, as stipulated in WHO methods for measuring medicine availability.^23^ Oral and intravenous formulations were assessed separately. Each survey questionnaire was country-specific and the number of antibiotics assessed varied slightly between countries. If the availability of a particular antibiotic was not assessed in a country, data for that antibiotic were classed as missing data. The availability of an antibiotic in a country was defined as the percentage of health facilities in that country where the antibiotic was available. The median and interquartile range (IQR) of the percentage availability across all countries were calculated. Availability is presented according to AWaRe categories (Table 1).

The use of antibiotics for treating particular illnesses in children was assessed in service provision assessment surveys that included observations of child consultations. Trained interviewers asked health-care providers (e.g. a medical doctor, nurse, nonphysician clinical specialist or midwife) about the children’s diagnoses and what treatment was prescribed or provided. Diagnoses were based on the children’s medical history and physical examinations, except for malaria, where the diagnosis was based on a rapid diagnostic test, blood smear microscopy or clinical findings, depending on the services available – details are provided in the online observation protocol.^24^

The percentage of children who were prescribed, or provided with, an antibiotic for each condition diagnosed was calculated for each survey country individually and overall. The diagnostic categories were: (i) pneumonia; (ii) asthma; (iii) other respiratory tract infection, including other upper respiratory infections and unknown respiratory illness; (iv) ear infection; (v) throat infection; (vi) diarrhoea; (vii) malaria; (vii) undifferentiated fever or measles; and (viii) any other illness. If a child was diagnosed with more than one condition, they were regarded as being diagnosed with the condition for which they were most likely to receive an antibiotic, this was determined using WHO’s 2017 *Model list of essential medicines for children*.^25^

The association between the availability and use of each antibiotic was assessed by multivariable logistic regression, which included adjustment for confounding variables, such as the child’s sex and age, the survey country and year, the type of facility, the condition diagnosed, the facility’s managing authority, the role of the health-care provider and season. Our study abided by WHO ethics and research committee rules and procedures on research involving human participants.

## Results

We identified 65 (38 service availability and readiness assessment and 27 service provision assessment) surveys conducted between 1997 and 2017. Although other surveys may have been carried out, we were not able to obtain either data or reports for our analysis. Of the 65, we excluded 3 because they were conducted between 1997 and 2000, 19 because more recent data were available for the country surveyed and 22 because no microdata were available (available in the data repository).^22^ The final sample included surveys from 20 locations (13 service provision assessment surveys and 8 service availability and readiness assessment surveys) conducted between 2004 and 2017, mainly in Africa. They covered a total of 13 561 health facilities (Table 3), of which 9111 (67.2%) were government facilities. The most common type of facility was the health centre, which comprised 39.1% (5302) of facilities. Overall, the surveys investigated the availability of 27 antibiotic formulations (19 access, 7 watch and 1 unclassified antibiotic); 17 were oral and 10 were intravenous. Ten of the service provision assessment surveys collected data on antibiotic use in a total of 22 699 children (Table 4): 99.4 % (21 604/21 715) of children whose ages were known were younger than 5 years of age.

**Table 3:** Health facilities, service provision assessment and service availability and readiness assessment surveys in low- and middle-income countries, 2004–2017

Location	Survey type (year)	No. of facilities surveyed (% of all facilities in location)^a^		No. of facilities (% of facilities surveyed)^b^						
				Type of facility				Managing authority		
				Hospital	Health centre	Clinic or dispensary		Government	Private	Not-for-profit organization
Bangladesh	SPA (2014)	1 596 (8.3)		185 (11.6)	541 (33.9)	870 (54.5)		1352 (84.7)	103 (6.5)	141 (8.8)
Benin	SARA (2014)	788 (55.0)		46 (5.8)	596 (75.6)	146 (18.5)		591 (75.0)	60 (7.6)	137 (17.4)
Democratic Republic of the Congo	SARA (2013)	1 555 (9.6)		485 (31.2)	706 (45.4)	364 (23.4)		872 (56.1)	248 (15.9)	433 (27.8)
Egypt^c^	SPA (2004)	659 (12.9)		81 (12.3)	373 (56.6)	205 (31.1)		559 (84.8)	0 (0.0)	100 (15.2)
Guyana	SPA (2004)	155 (47.5)		30 (19.4)	69 (44.5)	56 (36.1)		30 (19.4)	69 (44.5)	47 (30.3)
Haiti^c^	SPA (2013)	907 (100.0)		121 (13.3)	427 (47.1)	359 (39.6)		344 (37.9)	214 (23.6)	349 (38.5)
Kenya^c^	SPA (2010)	695 (11.2)		252 (36.3)	101 (14.5)	342 (49.2)		347 (49.9)	217 (31.2)	131 (18.8)
Malawi^c^	SPA (2013–2014)	1 060 (100.0)		119 (11.2)	484 (45.7)	457 (43.1)		509 (48.0)	252 (23.8)	229 (21.6)
Mauritania	SARA (2013)	232 (100.0)		37 (15.9)	123 (53.0)	72 (31.0)		163 (70.3)	61 (26.3)	8 (3.4)
Namibia^c^	SPA (2009)	411 (92.2)		45 (10.9)	47 (11.4)	319 (77.6)		306 (74.5)	49 (11.9)	42 (10.2)
Nepal^c^	SPA (2015)	992 (21.0)		270 (27.2)	247 (24.9)	475 (47.9)		775 (78.1)	139 (14.0)	78 (7.9)
Rwanda^c^	SPA (2017)	538 (96.9)		42 (7.8)	389 (72.3)	107 (19.9)		309 (57.4)	229 (42.6)	0 (0.0)
Senegal^c^	SPA (2017)	794 (21.1)		37 (4.7)	75 (9.4)	682 (85.9)		706 (88.9)	0 (0.0)	88 (11.1)
Sierra Leone	SARA (2016)	455 (36.0)		264 (58.0)	191 (42.0)	0 (0.0)		399 (87.7)	22 (4.8)	34 (7.5)
Somalia	SARA (2012)	149 (13.9)		11 (7.4)	73 (49.0)	65 (43.6)		144 (96.6)	0 (0.0)	3 (2.0)
Togo	SARA (2013)	100 (12.8)		32 (32.0)	39 (39.0)	29 (29.0)		75 (75.0)	9 (9.0)	13 (13.0)
Uganda^c^	SPA (2005)	491 (100.0)		119 (24.2)	372 (75.8)	0 (0.0)		351 (71.5)	140 (28.5)	0 (0.0)
United Republic of Tanzania^c^	SPA (2014–2015)	1 200 (17.7)		263 (21.9)	380 (31.7)	557 (46.4)		783 (65.2)	188 (15.7)	204 (17.0)
Zambia	SPA (2015)	424 (23.0)		101 (23.8)	0 (0.0)	52 (12.3)		305 (71.9)	0 (0.0)	119 (28.1)
Zanzibar	SARA (2012)	79 (29.9)		8 (10.1)	69 (87.3)	2 (2.5)		77 (97.5)	1 (1.3)	1 (1.3)
Zimbabwe	SARA (2015)	275 (25.2)		62 (22.6)	0 (0.0)	184 (66.9)		114 (41.5)	18 (6.5)	46 (16.7)
**Total**	**13 SPAs, 8 SARAs**	**13 561 (18.3)**		**2610 (19.3)**	**5302 (39.1)**	**5343 (39.4)**		**9111 (67.2)**	**2019 (14.9)**	**2203 (16.2)**

SARA: Service Availability and Readiness Assessment; SPA: Service Provision Assessment.

^a^ The total number of facilities in each location was based on the master facility list provided by health ministries.

^b^ Data on facility type and managing authority were not available for 306 and 222 facilities, respectively. Consequently, for some countries percentages do not total 100%.

^c^ Child consultations were observed in this survey.

**Table 4:** Child consultations observed, service provision assessment surveys in low- and middle-income countries, 2004–2017

Variable	No. (%)^a^											
	Country, year of survey											Total (*n* = 22 699)
	Egypt, 2004 (*n* = 2069)	Haiti, 2013 (*n* = 2 450)	Kenya, 2010 (*n* = 2047)	Malawi, 2013–2014 (*n* = 3 438)	Namibia, 2009 (*n* = 1 578)	Nepal, 2015 (*n* = 2 229)	Rwanda, 2017 (*n* = 1 756)	Senegal, 2017 (*n* = 1 064)	Uganda, 2005 (*n* = 1 112)	United Republic of Tanzania, 2014–2015 (*n* = 4 956)		
**Child’s age in years, mean (SD)**	1.8 (1.3)	1.7 (1.3)	1.9 (1.4)	1.8 (1.3)	1.9 (1.4)	1.9 (1.3)	1.8 (1.4)	1.6 (1.2)	1.6 (1.2)	1.7 (1.3)		1.8 (1.3)
**Male children**	1164 (56.3)	1180 (48.2)	1080 (52.8)	1719 (50.0)	830 (52.6)	1266 (56.8)	927 (52.8)	558 (52.4)	553 (49.8)	2562 (51.7)		11 839 (52.2)
**Male physicians**	1404 (67.9)	926 (37.8)	1109 (54.2)	2496 (72.6)	412 (26.1)	1738 (78.0)	904 (51.5)	497 (46.7)	647 (58.2)	3061 (61.8)		13 194 (58.1)
**Physician type^b^**												
Medical doctor	2069 (100)	1512 (61.7)	1197 (58.5)	55 (1.6)	61 (3.9)	341 (15.3)	120 (6.9)	105 (9.9)	5 (0.5)	344 (6.9)		5 809 (25.6)
Nurse or midwife	0 (0.0)	936 (38.2)	792 (38.7)	591 (17.2)	1508 (95.6)	183 (8.2)	1614 (92.3)	899 (84.5)	381 (34.4)	1130 (22.8)		8 034 (35.4)
Health-care assistant^c^	0 (0.0)	0 (0.0)	0 (0.0)	2787 (81.1)	9 (0.6)	1705 (76.5)	3 (0.2)	0 (0.0)	718 (64.9)	3477 (70.2)		8 699 (38.3)
**Condition diagnosed**												
Pneumonia	103 (5.0)	60 (2.4)	312 (15.2)	404 (11.8)	145 (9.2)	177 (7.9)	133 (7.6)	110 (10.3)	148 (13.3)	656 (13.2)		2 248 (9.9)
Ear infection	46 (2.2)	70 (2.9)	53 (2.6)	74 (2.2)	46 (2.9)	108 (4.8)	50 (2.8)	12 (1.1)	29 (2.6)	64 (1.3)		552 (2.4)
Throat infection	550 (26.6)	25 (1.0)	33 (1.6)	18 (0.5)	55 (3.5)	25 (1.1)	83 (4.7)	10 (0.9)	4 (0.4)	89 (1.8)		892 (3.9)
Asthma	0 (0.0)	46 (1.9)	42 (2.1)	23 (0.7)	11 (0.7)	9 (0.4)	6 (0.3)	17 (1.6)	24 (2.2)	32 (0.6)		210 (0.9)
Other respiratory tract infection	649 (31.4)	605 (24.7)	959 (46.8)	1222 (35.5)	769 (48.7)	456 (20.5)	888 (50.6)	331 (31.1)	525 (47.2)	1490 (30.1)		7 894 (34.8)
Malaria	0 (0.0)	106 (4.3)	298 (14.6)	184 (5.4)	18 (1.1)	5 (0.2)	39 (2.2)	12 (1.1)	135 (12.1)	363 (7.3)		1 160 (5.1)
Diarrhoea	489 (23.6)	387 (15.8)	170 (8.3)	262 (7.6)	185 (11.7)	245 (11.0)	231 (13.2)	132 (12.4)	97 (8.7)	634 (12.8)		2 832 (12.5)
Undifferentiated fever	59 (2.9)	131 (5.3)	47 (2.3)	38 (1.1)	41 (2.6)	355 (15.9)	184 (10.6)	123 (11.6)	102 (9.2)	438 (8.8)		1 518 (6.7)
Other diagnosis	71 (3.4)	835 (34.1)	0 (0.0)	303 (8.8)	109 (6.9)	586 (26.3)	93 (5.3)	248 (23.3)	26 (2.3)	414 (8.4)		2 685 (11.8)
No diagnosis	102 (4.9)	185 (7.6)	133 (6.5)	910 (26.5)	199 (12.6)	263 (11.8)	49 (2.8)	69 (6.5)	22 (2.0)	776 (15.7)		2 708 (11.9)
**Antibiotic prescribed**	1246 (60.2)	999 (40.8)	1506 (74.5)	2241 (65.2)	1173 (74.6)	879 (39.4)	1046 (60.5)	525 (49.3)	609 (54.8)	3372 (68.0)		13 638 (60.1)

SD: standard deviation.

^a^ All values in the table represent absolute numbers and percentages unless otherwise stated.

^b^ The total number of physician types may not equal the number of consultations observed because nonclinical personnel (such as laboratory officers) conducted the consultations in some cases.

^c^ The technical qualification of health-care assistants was country-specific; assistants included community health workers, medical assistants or officers and clinical assistants or officers.

### Antibiotic availability

**Fig. 1:**
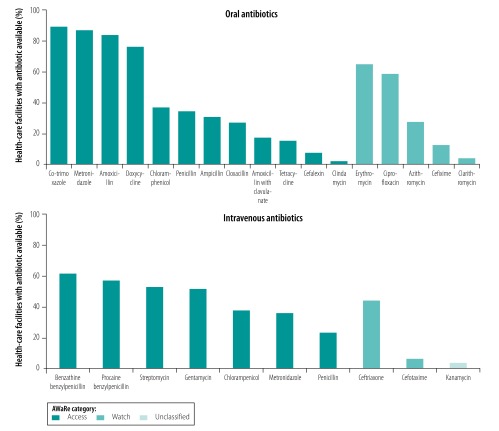
Antibiotic availability at health facilities, service provision assessment and service availability and readiness assessment surveys in low- and middle-income countries, 2004–2017

Notes: The bars represent the median availability across all countries surveyed. Antibiotics were classified using the World Health Organization’s AWaRe categories (Box 1).

The median availability of all antibiotics at all health facilities in the surveys was 48.9%. The access antibiotics were most often investigated in surveys and were the most widely available at health facilities. Although no access antibiotic was universally available, the median proportion of facilities across countries with co-trimoxazole, metronidazole and amoxicillin available was 89.5% (IQR: 12.6%), 87.1% (IQR: 15.9%) and 83.8% (IQR: 26.4%), respectively (Fig. 1). Some access antibiotics (i.e. ampicillin, cloxacillin, amoxicillin with clavulanate, tetracycline, cefalexin and clindamycin) were available in a median of 30.9% or fewer health facilities, although cefalexin and clindamycin were assessed in only six and five surveys, respectively (Table 5 available at: http://www.who.int/bulletin/volumes/98/3/19-241349, and data repository).^22^

**Table 5:** Antibiotic availability at health facilities, service provision assessment and service availability and readiness assessment surveys in low- and middle-income countries, 2004–2017

Type of antibiotic^a^	No. of health facilities with antibiotic available / no. of facilities providing information (%)																					Availability, median % (IQR)
	Location and year of survey^b^																					
	Bangladesh, 2014	Benin, 2014	Democratic Republic of the Congo, 2013	Egypt, 2004	Guyana, 2004	Haiti, 2013	Kenya, 2010	Malawi, 2013–2014	Mauritania, 2013	Namibia, 2009	Nepal, 2015	Rwanda, 2017	Senegal, 2017	Sierra Leone, 2016	Somalia, 2012	Togo, 2013	Uganda, 2005	United Republic of Tanzania, 2014–2015	Zambia, 2015	Zanzibar, 2012	Zimbabwe, 2015	
**Access (oral)**																						
Amoxicillin	1410/1506 (93.6)	731/770 (94.5)	1010/1254 (80.5)	345/464 (74.4)	82/110 (74.5)	781/887 (88.0)	389/640 (60.8)	857/964 (88.9)	153/164 (93.3)	326/389 (83.8)	846/918 (92.2)	405/486 (83.3)	325/367 (88.6)	34/449 (7.6)	35/56 (62.5)	3/99 (3.0)	200/479 (41.8)	1028/1170 (87.9)	348/401 (86.8)	31/75 (41.3)	1410/1506 (93.6)	83.8 (26.4)
Amoxicillin with clavulanate	295/1506 (19.6)	ND	ND	ND	ND	154/887 (17.4)	220/640 (34.4)	125/964 (13.0)	ND	31/389 (8.0)	224/918 (24.4)	95/486 (19.5)	73/367 (19.9)	ND	ND	ND	39/479 (8.1)	178/1170 (15.2)	53/399 (13.3)	ND	295/1506 (19.6)	17.4 (6.9)
Ampicillin	263/1506 (17.5)	541/773 (70.0)	1025/1314 (78.0)	178/461 (38.6)	37/110 (33.6)	257/887 (29.0)	123/640 (19.2)	70/964 (7.3)	130/159 (81.8)	66/389 (17.0)	137/918 (14.9)	345/486 (71.0)	304/367 (82.8)	302/447 (67.6)	47/71 (66.2)	23/99 (23.2)	147/479 (30.7)	361/1170 (30.9)	146/402 (36.3)	7/75 (9.3)	263/1506 (17.5)	30.9 (50.1)
Cefalexin	127/1506 (8.4)	ND	ND	ND	ND	ND	112/640 (17.5)	ND	ND	10/389 (2.6)	ND	5/486 (1.0)	ND	ND	ND	ND	32/479 (6.7)	ND	80/401 (20.0)	ND	127/1506 (8.4)	7.6 (14.9)
Chloramphenicol	ND	ND	ND	ND	ND	ND	147/640 (23.0)	ND	ND	45/389 (11.6)	374/918 (40.7)	247/486 (50.8)	ND	ND	ND	ND	158/479 (33.0)	ND	186/401 (46.4)	ND	ND	36.9 (23.4)
Clindamycin	ND	ND	ND	ND	ND	ND	60/640 (9.4)	ND	ND	32/389 (8.2)	ND	5/486 (1.0)	ND	ND	ND	ND	10/479 (2.1)	ND	6/401 (1.5)	ND	ND	2.1 (6.7)
Cloxacillin	268/1506 (17.8)	770/770 (100.0)	ND	ND	ND	ND	ND	ND	ND	ND	ND	ND	ND	ND	ND	ND	ND	ND	109/402 (27.1)	ND	268/1506 (17.8)	27.1 (82.2)
Co-trimoxazole	1313/1506 (87.2)	764/770 (99.2)	1103/1254 (88.0)	304/463 (65.7)	ND	730/887 (82.3)	580/640 (90.6)	906/964 (94.0)	148/163 (90.8)	372/389 (95.6)	815/918 (8.8)	438/486 (90.1)	249/367 (67.8)	274/449 (61.0)	48/56 (85.7)	99/99 (100.0)	380/479 (79.3)	1057/1170 (90.3)	301/402 (74.9)	68/75 (90.7)	1313/1506 (87.2)	89.5 (11.6)
Doxycycline	1113/1506 (73.9)	ND	ND	19/462 (4.1)	61/110 (55.5)	574/887 (64.7)	488/640 (76.2)	838/964 (86.9)	ND	333/389 (85.6)	408/918 (44.4)	389/486 (80.0)	299/367 (81.5)	ND	ND	ND	351 /479 (73.3)	893/1170 (76.3)	358/402 (89.1)	ND	1113/1506 (73.9)	76.3 (16.8)
Metronidazole	1422/1506 (94.4)	730/770 (94.8)	1044/1254 (83.3)	ND	80/110 (72.7)	641/887 (72.3)	425/640 (66.4)	862/964 (89.4)	145/161 (90.1)	349/389 (89.7)	882/918 (96.1)	424/486 (87.2)	297/367 (80.9)	ND	46/56 (82.1)	86/99 (86.9)	355/479 (74.1)	847/1170 (72.4)	361/402 (89.8)	ND	1422/1506 (94.4)	87.1 (15.9)
Penicillin	710/1506 (47.1)	ND	ND	30/463 (6.5)	ND	ND	55/640 (8.6)	133/964 (13.8)	ND	324/389 (83.3)	ND	349/486 (71.8)	ND	ND	ND	ND	105/479 (21.9)	ND	253/400 (63.2)	ND	710/1506 (47.1)	34.5 (56.3)
Tetracycline	502/1506 (33.3)	ND	ND	295/464 (63.6)	ND	156/887 (17.6)	84/640 (13.1)	66/964 (6.8)	ND	6/389 (1.5)	403/918 (43.9)	111/486 (22.8)	11/367 (3.0)	ND	ND	ND	45/479 (9.4)	77/1170 (6.6)	72/402 (17.9)	ND	502/1506 (33.3)	15.4 (21.4)
**Access (intravenous)**																						
Benzathine benzylpenicillin	136/1506 (9.0)	685/716 (95.7)	734/1314 (55.9)	322/463 (69.5)	37/110 (33.6)	266/887 (30.0)	482/640 (75.3)	836/964 (86.7)	85/157 (54.1)	276/389 (71.0)	ND	416/486 (85.6)	273/367 (74.4)	18/447 (4.0)	43/71 (60.6)	5/99 (5.1)	267/479 (55.7)	857/1170 (73.2)	ND	46/75 (61.3)	136/1506 (9.0)	61.3 (41.7)
Chloramphenicol	ND	69/770 (9.0)	ND	ND	ND	ND	276/640 (43.1)	ND	ND	36/389 (9.3)	ND	234/486 (48.1)	ND	ND	ND	ND	256/479 (53.4)	ND	128/402 (31.8)	ND	ND	37.5 (38.9)
Gentamycin	195/1506 (12.9)	660/773 (85.4)	300/1314 (22.8)	219/464 (47.2)	40/110 (36.4)	254/887 (28.6)	503/640 (78.6)	829/964 (86.0)	121/159 (76.1)	119/389 (30.6)	669/918 (72.9)	266/496 (54.7)	301/367 (82.0)	446/451 (98.9)	47/71 (66.2)	84/99 (84.8)	233/479 (48.6)	591/1170 (50.5)	242/401 (60.3)	10/75 (13.3)	195/1506 (12.9)	56.6 (42.2)
Metronidazole	225/1506 (14.9)	ND	ND	308/464 (66.4)	ND	148/887 (16.7)	ND	106/964 (11.0)	ND	ND	328/918 (35.7)	ND	239/367 (65.1)	ND	ND	ND	ND	443/1170 (37.9)	ND	ND	225/1506 (14.9)	35.7 (50.2)
Penicillin	93/1506 (6.2)	ND	ND	ND	ND	247/887 (27.8)	ND	478/964 (49.6)	ND	ND	71/918 (7.7)	ND	67/367 (18.3)	ND	ND	ND	ND	738/1170 (63.1)	ND	ND	93/1506 (6.2)	23.1 (41.9)
Procaine benzylpenicillin	ND	565/770 (73.4)	641/1254 (51.1)	244/464 (52.6)	47/110 (42.7)	ND	180/640 (28.1)	ND	52/163 (31.9)	97/389 (24.9)	ND	403/486 (82.9)	ND	84/447 (18.8)	ND	84/99 (84.8)	347/479 (72.4)	ND	381/402 (94.8)	70/75 (93.3)	ND	52.6 (51.0)
Streptomycin	114/217 (52.5)	110/322 (34.2)	374/578 (64.7)	94/464 (20.3)	ND	107/269 (39.8)	ND	68/384 (17.7)	24/48 (50.0)	ND	ND	ND	47/367 (31.1)	433/446 (97.1)	11/13 (84.6)	29/36 (80.6)	ND	232/569 (40.8)	211/402 (52.5)	ND	114/217 (52.5)	51.2 (46.4)
**Watch (oral)**																						
Azithromycin	406/1506 (27.0)	685/773 (88.6)	374/1314 (28.5)	ND	ND	229/887 (25.8)	ND	375 /964 (38.9)	19/158 (12.0)	ND	311/918 (33.9)	ND	14/367 (4.8)	325/447 (72.7)	30/71 (42.3)	6/99 (6.1)	ND	335/1170 (28.6)	ND	10/75 (13.3)	406/1506 (27.0)	27.7 (8.1)
Cefixime	309/1506 (20.5)	430/773 (55.6)	505/1314 (38.4)	ND	ND	72/887 (8.1)	ND	40/964 (4.1)	20/159 (12.6)	ND	233/918 (25.4)	ND	77/367 (21.0)	28/447 (6.3)	18/71 (25.4)	ND	ND	100/1170 (8.5)	ND	1/75 (1.3)	309/1506 (20.5)	14.5 (12.9)
Ciprofloxacin	883/1506 (58.6)	701/770 (91.0)	833/1254 (66.4)	18/461 (3.9)	48/110 (43.6)	545/887 (61.4)	413/640 (64.5)	528/964 (54.8)	65/162 (40.1)	340/389 (87.4)	301/918 (32.8)	360/486 (74.1)	324/367 (88.3)	146/449 (32.5)	27/56 (48.2)	77/99 (77.8)	311/479 (64.9)	936/1170 (80.0)	212/402 (52.7)	9/75 (12)	883/1506 (58.6)	58.6 (34.0)
Clarithromycin	ND	ND	ND	ND	ND	ND	80/640 (12.5)	ND	ND	16/389 (4.1)	ND	8/486 (1.6)	ND	ND	ND	ND	10/479 (2.1)	ND	17/402 (4.2)	ND	ND	4.1 (2.1)
Erythromycin	260/1506 (17.3)	416/596 (69.8)	ND	97/464 (20.9)	71/110 (64.5)	578/887 (65.2)	407/640 (63.6)	840/964 (87.1)	ND	323/389 (83.0)	113/918 (12.3)	399/486 (82.1)	208/367 (56.7)	ND	ND	ND	229/479 (47.8)	916/1170 (78.3)	333/XXX (82.8)	ND	260/1506 (17.3)	64.9 (34.3)
**Watch (intravenous)**																						
Cefotaxime	ND	ND	ND	ND	ND	ND	41/640 (6.4)	ND	ND	13/389 (3.3)	ND	37/486 (7.6)	ND	ND	ND	ND	4/479 (0.8)	ND	56/399 (14.0)	ND	ND	6.4 (4.3)
Ceftriaxone	288/1506 (19.1)	418/770 (54.3)	578/1274 (46.1)	20/462 (4.3)	ND	265/887 (29.9)	281/640 (43.9)	484/964 (50.2)	29/162 (17.9)	309/389 (79.4)	214/918 (23.3)	33/486 (6.8)	248/367 (67.6)	397/449 (88.4)	32/56 (57.1)	43/99 (43.4)	95/479 (19.8)	765/1170 (65.4)	42/402 (10.4)	19/75 (25)	288/1506 (19.1)	43.7 (36.2)
**Unclassified**																						
Kanamycin (intravenous)	ND	ND	ND	ND	ND	ND	24/640 (3.8)	ND	ND	24/389 (6.2)	ND	4/486 (0.8)	ND	ND	ND	ND	0/479 (0.0)	ND	99/402 (24.6)	ND	ND	4 (5.3)

IQR: interquartile range; ND: not determined.

^a^ Antibiotics were classified using the World Health Organization’s AWaRe categories. See Box 1.

^b^ A service provision assessment survey or service availability and readiness assessment survey was carried out in location (Table 3).

The surveys assessed seven watch antibiotics, which were less frequently available than access antibiotics. The most widely available watch antibiotic was erythromycin, which had a median overall availability of 65% (IQR: 34; Fig. 1, Table 5, and data repository).^22^ Across all AWaRe categories, there were some large variations between and within countries; for example, the proportion of facilities with benzathine benzylpenicillin (an access antibiotic) in stock ranged from 4% in Sierra Leone to 96% in Benin (Table 5). In total, 17 access and watch antibiotics were, on average, stocked by fewer than 50% of facilities.

### Antibiotic use

**Fig. 2:**
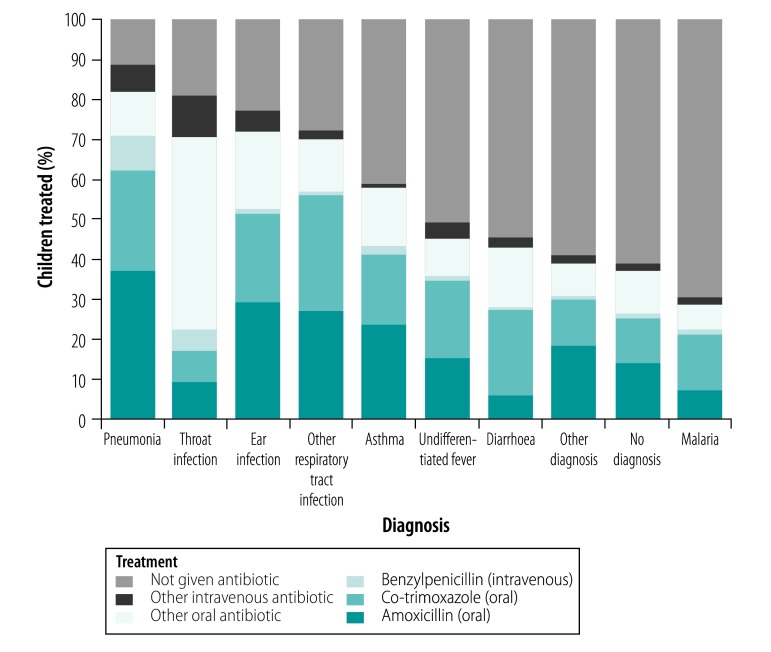
Proportion of children prescribed antibiotics, by antibiotic type and diagnosis, service provision assessment surveys in 10 low- and middle-income countries, 2004–2017

Notes: Percentages represented are the median of prescribed antibiotics across all countries surveyed. Diagnoses were made by health-care providers in consultations observed during service provision assessment surveys. For Rwanda and Uganda, which recorded amoxicillin and co-trimoxazole in a single category, we assigned all entries in that category to co-trimoxazole because the availability of co-trimoxazole was greater than that of amoxicillin in both countries. Other respiratory tract infection includes unspecified upper respiratory infection and other unknown respiratory illnesses. Undifferentiated fever includes cases where the cause of the fever was not known or the diagnosis was measles. Diarrhoea includes diarrhoea and dysentery. An observation was classified as no diagnosis when no diagnosis was recorded but treatment was still given. The data for each country is available from the corresponding author. Countries included: Egypt, Haiti, Kenya, Malawi, Namibia, Nepal, Rwanda, Senegal, Uganda and United Republic of Tanzania.

Overall, 60.1% (13 638/22 699) of children whose consultations were observed were prescribed an antibiotic (Table 4); of the 13 638, 4724 (34.6%) received co-trimoxazole, 4525 (33.2%) received amoxicillin and 416 (3.1%) received intravenous benzylpenicillin (all access antibiotics). Children diagnosed with a respiratory condition were most likely to be prescribed an antibiotic, the proportion was 76.1% (8972/11 796). Specifically, 88.9% (1998/2248), 80.9% (722/892) and 72.2% (5701/7894) of children with pneumonia, throat infections and other respiratory tract infections, respectively, received an antibiotic (Table 6). In addition, an antibiotic was prescribed for 50.1% (760/1518) of undifferentiated fever cases, 45.7% (1293/2832) of diarrhoea cases and 30.3% (352/1160) of malaria cases. Amoxicillin and co-trimoxazole were the most commonly prescribed antibiotics for all diagnoses, except throat infection (Fig. 2). Multivariable logistic regression showed that the availability of an antibiotic was significantly associated with its use: the odds that amoxicillin would be used if it were available was 1.40 (95% confidence interval, CI: 1.26–1.55). The corresponding odds was 1.38 (95% CI: 1.12–1.71) for benzylpenicillin, 1.94 (95% CI: 1.63–2.29) for co-trimoxazole, 1.24 (95% CI: 0.98–1.56) for all other intravenous antibiotics and 1.02 (95% CI: 0.88–1.18) for all other oral antibiotics.

**Table 6:** Proportion of children prescribed an antibiotic during consultations, by diagnosis, service provision assessment surveys, 2004–2017

Diagnosis	No. (%) of children prescribed an antibiotic^a^										
	Country (year) of survey										Total
	Egypt (2004)	Haiti (2013)	Kenya (2010)	Malawi (2013–14)	Namibia (2009)	Nepal (2015)	Rwanda (2017)	Senegal (2017)	Uganda (2005)	United Republic of Tanzania (2014–2015)	
Pneumonia	87 (84.5)	40 (66.7)	300 (96.2)	394 (97.5)	134 (92.4)	109 (61.6)	111 (83.5)	75 (68.2)	118 (79.7)	630 (96.0)	1 998 (88.9)
Ear infection	43 (93.5)	56 (80.0)	49 (92.5)	72 (97.3)	38 (82.6)	53 (49.1)	37 (74.0)	10 (83.3)	19 (65.5)	50 (78.1)	427 (77.4)
Throat infection	467 (84.9)	18 (72.0)	29 (87.9)	16 (88.9)	51 (92.7)	3 (12.0)	56 (67.5)	5 (50.0)	2 (50.0)	75 (84.3)	722 (80.9)
Asthma	0 (0.0)	26 (56.5)	32 (76.2)	17 (73.9)	8 (72.7)	0 (0.0)	3 (50.0)	6 (35.3)	12 (50.0)	20 (62.5)	124 (59.0)
Other respiratory tract infection	403 (62.1)	253 (41.8)	814 (84.9)	1092 (89.4)	624 (81.1)	146 (32.0)	614 (69.1)	179 (54.1)	345 (65.7)	1231 (82.6)	5 701 (72.2)
Malaria	0 (0.0)	40 (37.7)	98 (32.9)	43 (23.4)	8 (44.4)	0 (0.0)	15 (38.5)	2 (16.7)	33 (24.4)	113 (31.1)	352 (30.3)
Diarrhoea	179 (36.6)	154 (39.8)	111 (65.3)	99 (37.8)	117 (63.2)	60 (24.5)	103 (44.6)	34 (25.8)	37 (38.1)	399 (62.9)	1 293 (45.7)
Undifferentiated fever	32 (54.2)	53 (40.5)	16 (34.0)	26 (68.4)	23 (56.1)	116 (32.7)	69 (37.5)	77 (62.6)	30 (29.7)	318 (72.6)	760 (50.1)
Other diagnosis	17 (23.9)	323 (38.7)	0 (0.0)	124 (40.9)	47 (43.1)	288 (49.1)	35 (37.6)	108 (43.5)	11 (42.3)	207 (50.0)	1 160 (43.2)
No diagnosis given	18 (17.6)	36 (19.5)	77 (57.9)	358 (39.3)	127 (63.8)	104 (39.5)	21 (42.9)	29 (42.0)	2 (9.1)	329 (42.4)	1 101 (40.7)
**Total**	1246 (60.2)	999 (40.8)	1526 (74.5)	2241 (65.2)	1177 (74.6)	879 (39.4)	1064 (60.6)	525 (49.3)	609 (54.8)	3372 (68.0)	13 638 (60.1)

^a^ Percentages were calculated using the number of children diagnosed with the condition in each country given in Table 4.

## Discussion

Using the data from service provision assessment and service availability and readiness assessment surveys have great potential for informing countries about the pattern of antibiotic use at health facilities. In addition, the data can also be used by antimicrobial resistance coordination committees. As the majority of facilities surveyed in our study were health centres, clinics or dispensaries, it is appropriate that access antibiotics were more widely available than watch antibiotics. Of access antibiotics, several were available at most facilities, such as amoxicillin and co-trimoxazole. However, other access antibiotics were much less available: gentamycin, which is used for treating neonatal sepsis and other severe infections,^26^ was available at only 56.6% of facilities. Moreover, most facilities had shortages of watch antibiotics, which have a key therapeutic role in some infections. Although the quantity required may be small, they should be available at all health facilities.

Overall, we found that 60.1% of children whose consultations were observed were prescribed an antibiotic. Although there may be valid reasons for treating conditions, such as upper respiratory tract infection and diarrhoea, the high percentage of prescribing suggests antibiotics were being used inappropriately, as has been observed both anecdotally and in other studies.^27^^–^^29^ Among diagnoses, respiratory conditions had the highest percentage of antibiotic prescription. However, many children were prescribed antibiotics for conditions for which they are not usually indicated, including undifferentiated fever, diarrhoea and malaria.

Globally there is considerable debate about the importance of access to antibiotics, but this frequently focuses on national supplies. Some studies have used pharmaceutical sales data, which reflect the antibiotic consumption of whole countries rather than individuals or communities.^5^^,^^30^^,^^31^ Nevertheless, despite the different data sources used, the proportion of prescribed antibiotics that were access antibiotics was broadly similar across paediatric studies. One international study found that 76.0% of all antibiotics used were access antibiotics (compared with 72.4% in our study) and 30.7% were amoxicillin (compared with 33.9% in our study).^30^ However, there are inconsistencies between health facility surveys and pharmaceutical sales studies because the antibiotics with the highest sales are not always available at health facilities. For example, in our study, amoxicillin with clavulanic acid was available at only 17.4% of health facilities, whereas recent global pharmaceutical sales data indicate it is used in almost equal amounts to amoxicillin, which was available at 83.8% of facilities in our study.^31^ Similarly, cefixime is also one of the most commonly used antibiotics according to pharmaceutical sales, but was available at only 12.9% of facilities.^31^ These discrepancies suggest there may be a divergence between supply and use in some countries. There might be differences in prescribing patterns between health facilities and other vendors who were not covered in our study, but whose antibiotic sales were reflected in pharmaceutical data.

The lack of high-quality data at the community level presents a barrier to understanding antibiotic access and use. One systematic analysis of antibiotic consumption in countries in the WHO African Region found that studies were frequently limited by their small sample size, a lack of adherence to WHO recommendations on reporting medicines and poor reporting of study details,^32^ which illustrates the difficulty of obtaining meaningful data in low- and middle-income countries. Despite methodological difficulties, community studies in India, Nepal, Viet Nam and in countries in sub-Saharan Africa have reported high and possibly inappropriate percentages of antibiotic prescriptions in the range of 41.0–85.0%, similar to our finding in children.^33^^–^^39^ The main advantage of these analyses is that they can identify the diseases for which antibiotics are most commonly used and they consistently showed most antibiotic prescriptions were for respiratory conditions.^33^^,^^34^^,^^36^^,^^37^^,^^40^^,^^41^ They also documented high antibiotic use by individuals with malaria, despite rapid malaria diagnostic tests being available. The antibiotic prescription rates for diarrhoea and undifferentiated fever reported in several low- and middle-income countries were comparable with our findings.^34^^,^^39^^,^^42^^–^^46^

In our study, amoxicillin accounted for 33.9% of antibiotics prescribed to children, which was low relative to other community studies where amoxicillin made up over half of prescriptions.^34^^,^^40^^,^^47^^,^^48^ We found the variation in availability between countries was greater for amoxicillin than co-trimoxazole, which is often widely distributed within countries because HIV programmes have made substantial investments in supply chain management to ensure its availability for prophylaxis.^49^^,^^50^ However, the ready availability of co-trimoxazole can result in it being heavily prescribed even when inappropriate.^37^^,^^47^ As expected, availability was correlated with use, though the association was weak, probably because patients were instructed to buy specific medicines elsewhere if they were not available at a facility.

One strength of our study is that the surveys were reliably conducted in many countries, had large sample sizes and adopted a standard approach.^20^ Both assessment surveys are well suited to assessing all aspects of health-care provision, and the physical and human resources required. Although their total cost is high, these surveys offer a more efficient and cost–effective way of obtaining basic information on antibiotic consumption than specific surveys. Moreover, they can help monitor the actions taken to manage antimicrobial resistance both nationally and globally.

There are some limitations, however. First, our study was primarily an exploratory analysis of the usefulness of health facility surveys in low- and middle-income countries. Second, the survey data did not cover drug sources, such as local pharmacies or informal providers and not all antibiotics were included (e.g. no reserve antibiotics were monitored). Third, the availability of a medication may not correlate with its use because: (i) some countries use drug availability as a performance indicator, which may encourage suppliers to keep key medicines in stock instead of dispensing them; and (ii) the cost of an antibiotic (which was not recorded in surveys) may have been high enough to prevent individuals accessing it. Fourth, surveys were cross-sectional and thus reflected the status of facilities on one specific day, which may limit the generalizability of a survey’s findings beyond the specific country and year in which it was conducted. In particular, as some surveys were conducted over 10 years ago (i.e. in Egypt, Guyana and Uganda), recent antibiotic availability may have been underestimated. Finally, health workers are more likely to prescribe in accordance with guidelines when being observed.

More surveys are planned and underway. Future surveys will also collect information on the price of essential medicines to patients. Although assessments of facilities may not be able to provide detailed information on the formulation of drugs or on prescribing behaviour, they will continue to give insights into antibiotic availability and use in primary and secondary care, where monitoring capacity is limited but antibiotic use is greatest. Future surveys would benefit from the inclusion of standard questions on antibiotics based on AWaRe categories. As data from more surveys become available, future research will be able to monitor changing patterns of use.

This study of service provision assessment and service availability and readiness assessment surveys of health facilities in low- and middle-income countries demonstrated that more data on antibiotic availability and use are available than previously reported. These data can help countries evaluate the risk of antimicrobial resistance. Both surveys provide an important and expanding resource that can be used to improve understanding of local and global antibiotic consumption patterns, without the need for collecting new data. Our study found that first-line access antibiotics were unavailable at many health facilities in some countries, investment in antibiotic supply chain management is therefore needed. We also found that antibiotics were used extensively in primary care, often for conditions for which they are not usually indicated.
